# Evaluation of future estuarine floods in a sea level rise context

**DOI:** 10.1038/s41598-022-12122-7

**Published:** 2022-05-16

**Authors:** Carina Lurdes Lopes, Magda Catarina Sousa, Américo Ribeiro, Humberto Pereira, João Pedro Pinheiro, Leandro Vaz, João Miguel Dias

**Affiliations:** CESAM—Centre for Environmental and Marine Studies, Department of Physics, University of Aveiro, 3810-193 Aveiro, Portugal

**Keywords:** Projection and prediction, Natural hazards

## Abstract

Reliable predictions of future inundation extent within estuaries require a precise evaluation of future extreme sea levels and the application of accurate numerical models that account for the physical processes driving estuarine hydrodynamics. In this study, a methodology that integrates the estimation of local extreme sea levels with high-resolution numerical modeling was applied to assess the future inundation extent in five estuarine systems located on the Portuguese Coast. The main findings obtained were compared with available results from the popular bathtub approach, that disregards the physical processes driving estuarine hydrodynamics and therefore provide imprecise predictions of inundation extent and associated socio-economic impacts. The inundation extent is revealed to be highly dependent on the extreme sea levels and on the estuarine geomorphology, which controls the propagating long-wave. As the long-wave height is highly attenuated within estuaries that have adjacent low-lying areas, restricted inlets, or extensive tidal flats, the results of this study revealed that the extent of inundation is considerably smaller than that obtained by the bathtub approach. The uncertainties associated with mean sea level rise and the estuarine geomorphological evolution constitute the greatest difficulty in assessing the extent of flooding, posing major challenges to the efficient and sustainable management of estuaries.

## Introduction

Estuaries are coastal systems at the interface between the ocean, land and atmosphere that are constantly adapting to oceanographic, terrestrial and atmospheric conditions. Estuaries are extremely productive, supporting high biodiversity and attracting people to develop economic activities, including agriculture, industry, commerce and tourism^1–3^. Estuaries are particularly vulnerable to rising sea levels due to their proximity to the sea and their extensive adjacent floodplains often densely populated. It is estimated that 100,000 people in the EU are exposed to coastal flooding every year and that coastal flooding causes average damage of 1.4 billion € across Europe annually^4^. Moreover, projections indicate that in the absence of adaptation measures, between 1.6 and 3.9 million people will be affected by coastal floods by the end of the century, and the damage can reach between 210 billion € and 1.3 trillion €^4^.

Coastal inundations occur during extreme sea levels caused by a combination of tide, storm surge, wind-generated waves, and mean sea level^5^. For a correct evaluation of the inundation extent in estuaries, it is essential to accurately know the magnitude of these forcing and the morphological features of the estuarine regions^6–8^. To date, two approaches are often applied for estuarine inundation assessment: (1) bathtub; and (2) numerical modeling^9,10^. The bathtub approach roughly assumes that all regions below a certain water level are flooded if they have hydrological connectivity with other inundated zones, neglecting protection measures and physical processes that determine water level variations (e.g. bottom friction, flood protection barriers, flood duration, etc.)^10–13^. Because of this, bathtub approaches tend to exacerbate the inundation extent^11,14–16^ and consequently socio-economic damages^17^. Despite the limitations, bathtub models are easily implemented and adequate to assess coastal flood risk globally, providing crucial guidance in identifying areas vulnerable to coastal inundation^18,19^. However, on a local scale, the bathtub approach often fails in correctly predicting inundation extent since this approach does not account for water levels attenuation or amplification depending on the morphology of the estuary^20^. Hanslow et al.^20^ verified that this effect is particularly important in tidal lakes and lagoons, where water levels significantly attenuate during inland water propagation. In addition, Didier et al.^10^, Neumann and Ahrendt^21^ and Kumbier et al.^22^ compared flood mapping using both methods in Lower St. Lawrence estuary (Canada), Kiel Fjord (Germany) and estuaries in the South of Australia, respectively, and concluded that the performance of numerical models exceeds the bathtub approach.

For the Portuguese Coast, no studies have been identified comparing the performance of both methods, however, it is evident that the flood extent obtained through numerical modeling for the Tagus estuary^23^ and Ria de Aveiro^24,25^ are considerably lower than those obtained by Antunes et al.^26^ and Kulp en Strauss^27^ using the bathtub method. Despite these limitations, flood maps derived by previous authors were made available to the public through web GIS platforms (http://www.snmportugal.pt/index_EN.html; https://coastal.climatecentral.org/map/). Moreover, the flood maps obtained by Kulp and Strauss^27^ were widely disseminated by the Portuguese media in October and November 2019, and again recently, after the release of the 6th IPCC report. The exaggerated results of these models alarmed the public for the total or partial submersion of some important Portuguese cities (Lisbon, Aveiro, Figueira da Foz, etc.) by 2030, although some important technical details that support the determination of the flood maps have been omitted (e.g. frequency and duration of flood events, the existence of flood barriers, likely scenarios, etc.). Considering that flood mapping and flood communication directly influences flood risk perception^28–30^, it is fundamental to deliver reliable high resolution flood maps and support the translation of the scientific projections and data to the public. Otherwise, the disclosure of exaggerated projections has counterproductive effects, as it discredits the results of science and demobilizes the public about the relevance of climate change, since, due to their knowledge of the local reality, they do not consider the scenarios presented as possible. For these reasons, it is essential to perform a reliable assessment of the flood extent in estuaries and disseminate these results, to promote the literacy of the public and to make the results scientifically credible, while providing tools for public managers to make informed decisions.

**Figure 1:**
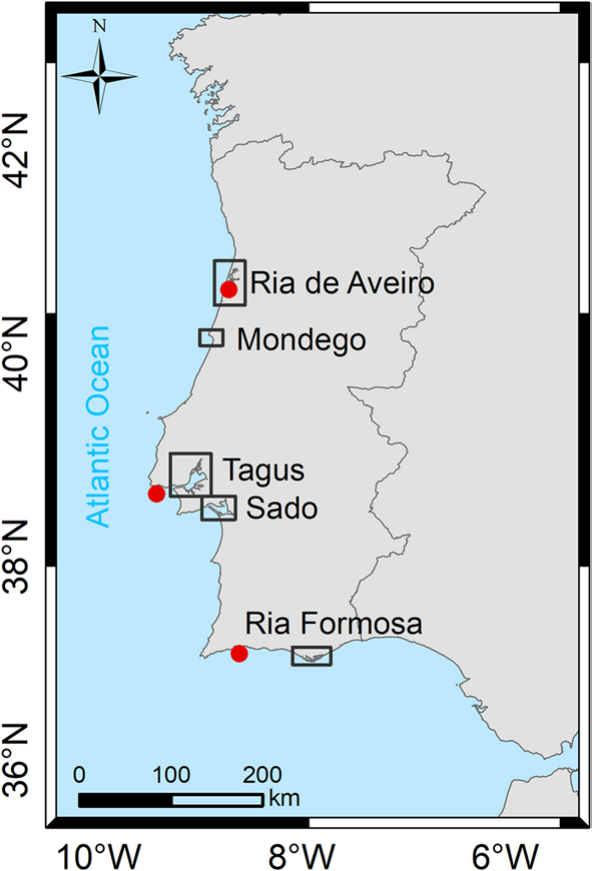
Map of Portugal depicting the location of the estuarine systems under study. The red dots show the location of tidal gauges whose elevation data was used to determine extreme levels (from north–south: Aveiro, Cascais and Lagos). This map was created with Esri ArcGIS 10.8 software (https://www.esri.com/en-us/home).

This study addresses these concerns and aims to highlight the importance of using high-resolution local numerical models and local forcing estimates for a reliable evaluation of future inundations in estuaries by analyzing the case of Portugal in a sea level rise context. The sea level oscillations at the Portuguese Coast were analyzed, and extreme sea level scenarios were defined for the future climate. Additionally, numerical applications for the five Portuguese estuarine systems (Fig. 1) most threatened by mean sea level rise were developed, validated, and applied to simulate inundation extent and the variations in water levels under future scenarios. The variations in the water level range were analyzed, and the processes affecting the long-wave propagation within the different estuaries were identified. Furthermore, the predicted inundation extent was integrated into Geographical Information Systems (GIS) and intersected with population and land use information, and the inhabitants and land surface area directly affected by inundation events were assessed and analyzed.

## Results

### Extreme sea levels

Results of extreme sea levels (Table 1) show distinct patterns for the Portuguese West (Aveiro and Cascais) and South Coasts (Lagos), highlighting a decrease from north to south. In Aveiro, extreme sea levels are slightly higher than in Cascais (0.03 m and 0.1 m, for 10 and 100 years return periods, respectively), denoting a small variability of extreme sea levels along the West Coast. Lagos (on the South Coast) presents the lowest extreme sea levels, which can be more than 0.30 m lower than those obtained for the West Coast. The differences between the west and south coasts are mainly due to the storm surge regime since the tides are very similar in the three locations (levels of Mean High Water Springs (MHWS) vary from 3.3 m in Aveiro and Cascais to 3.2 m in Lagos). This fact shows that storm surges are more severe on the West Coast and that the maximum height of frequent events (2 years) can be up to 0.20 m higher in the west than in the south.

**Table 1:** Extreme sea levels (m) relative to Chart Datum.

	2*	10	25	50	100*
Aveiro	3.96	4.25	4.38	4.47	4.55
Cascais	3.96	4.22	4.32	4.39	4.45
Lagos	3.63	3.96	4.09	4.18	4.29

The asterisk represents the return periods considered in flood modeling.

**Figure 2:**
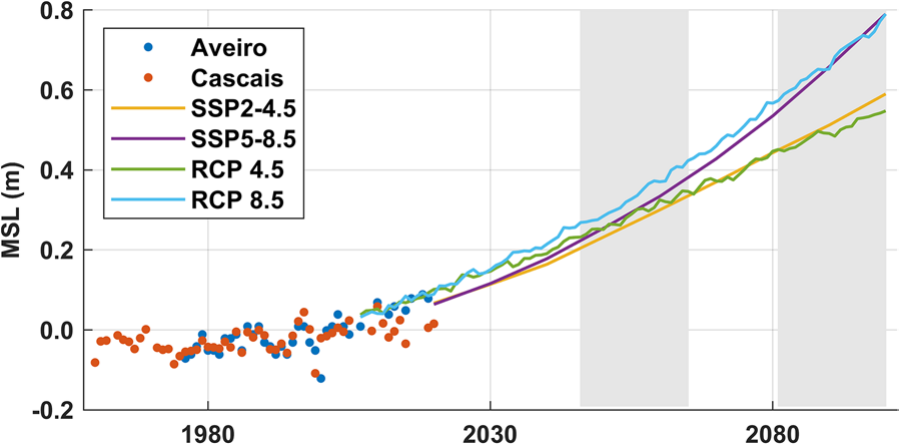
Mean sea level changes on the Portuguese Coast. The points represent observations and the lines projections. Observations and RCP scenarios are annual mean values relative to 1986–2005 and SSP scenarios are decadal mean values relative to 1995–2014. Climate induced mean sea level changes were evaluated by analyzing the sea level projection data from the IPCC climate projections (AR5 and AR6) for the Portuguese Coast.

Figure 2 presents annual mean sea levels derived from the tidal gauges of Aveiro and Cascais and the local projections for the twenty-first century based on IPCC climate projections (see “Methods” section). It should be noted that the Lagos data were discarded when analyzing mean sea levels since records present several problems of calibration and vertical referencing after 2000. The observed mean sea levels in Cascais are slightly higher than in Aveiro, although the variation patterns are similar in both locations evidencing a tendency for mean sea level rise. It should be highlighted that projections agree with observations for the period of coinciding data (2005–2019). Climate projections for RCP 4.5 and 8.5 are similar until 2030, but after 2030 the estimates diverge, with RCP 8.5 presenting the highest values. In fact, the mean sea level change projections (Table 2) for 2045–2065 only differ by 0.05 m between RCP 4.5 and 8.5. However, for 2081–2100 the estimate based on RCP 8.5 (0.68 m) is 0.2 m higher than that obtained for RCP 4.5 (0.50 m). The projections for AR5 (RCP 4.5 and RCP 8.5) and AR6 (SSP2-4.5 and SSP5-8.5) follow similar rise patterns. Before 2070, projections for RCP 4.5 are slightly higher than for SSP2-4.5, and after that, projections for SSP2-4.5 slightly exceed those for RCP 4.5. The same is observed when comparing SSP5-8.5 with RCP 8.5, but in this case the intersection of both curves occurs in 2090. The projections of mean sea level change (Table 2) confirm that the differences between RCP 4.5 and SSP2-4.5 and between RCP 8.5 and SSP5-8.5 are very low, reaching at most 0.04 m. The most pessimistic sea level change projections were obtained under scenario RCP 8.5, which will be considered hereafter in flood modeling.

**Table 2:** Mean sea level change (m) projections for the Portuguese Coast.

	RCP 4.5	RCP 8.5*	SSP2-4.5	SSP5-8.5
2046–2065	0.29	0.34	0.27	0.30
2081–2100	0.50	0.68	0.52	0.67

The asterisk represents the scenarios considered in flood modeling.

### Inundation assessment

The extreme sea levels for return periods of 2 and 100 years were combined with projections of mean sea level change by 2046–2065 and 2081–2100 under the RCP 8.5 (which present the highest mean rises) to define four flood-inducing scenarios for each location (Aveiro, Cascais and Lagos). These extreme sea levels were then used as oceanic boundary conditions in the numerical simulations attending to the proximity of the tidal gauges to the estuaries.

**Figure 3:**
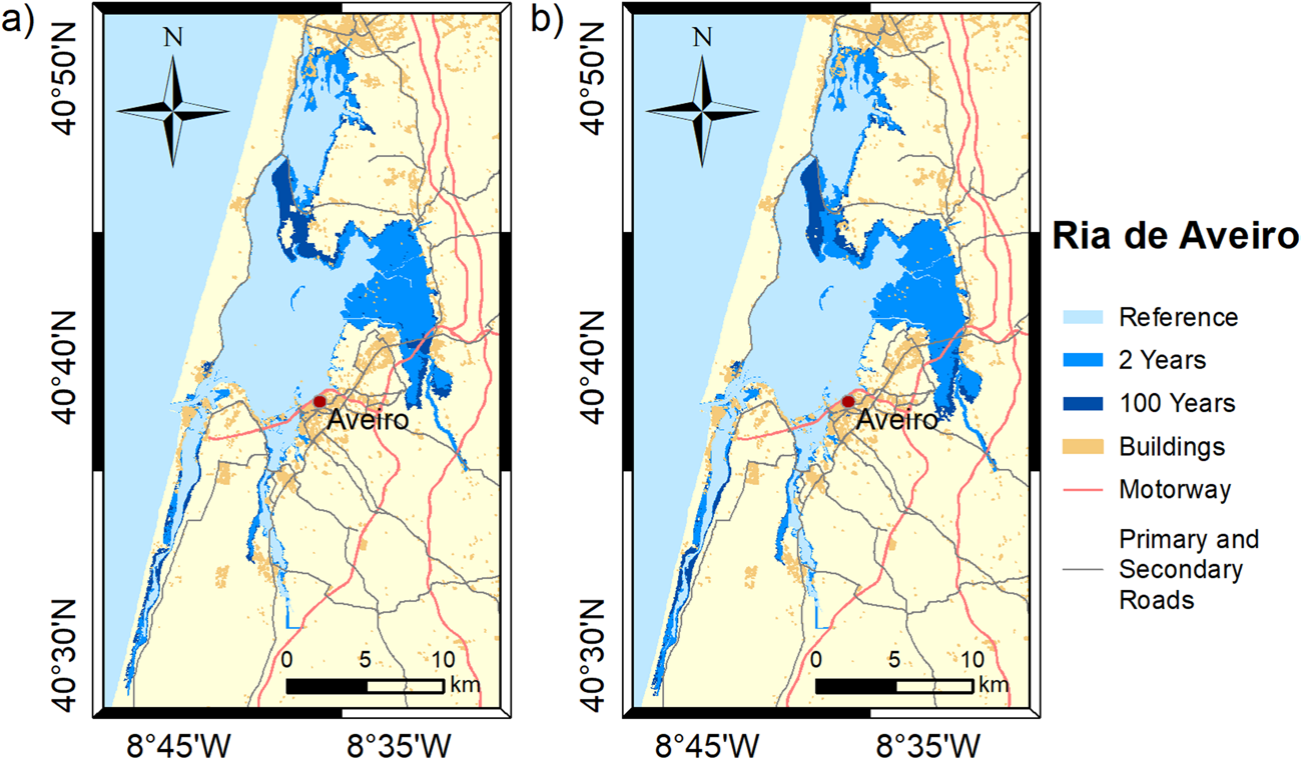
Inundation extents induced by extreme sea levels with return periods of 2 and 100 years on the Ria de Aveiro in: (**a**) 2046–2065; and (**b**) 2081–2100. These maps were created with Esri ArcGIS 10.8 software (https://www.esri.com/en-us/home).

**Figure 4:**
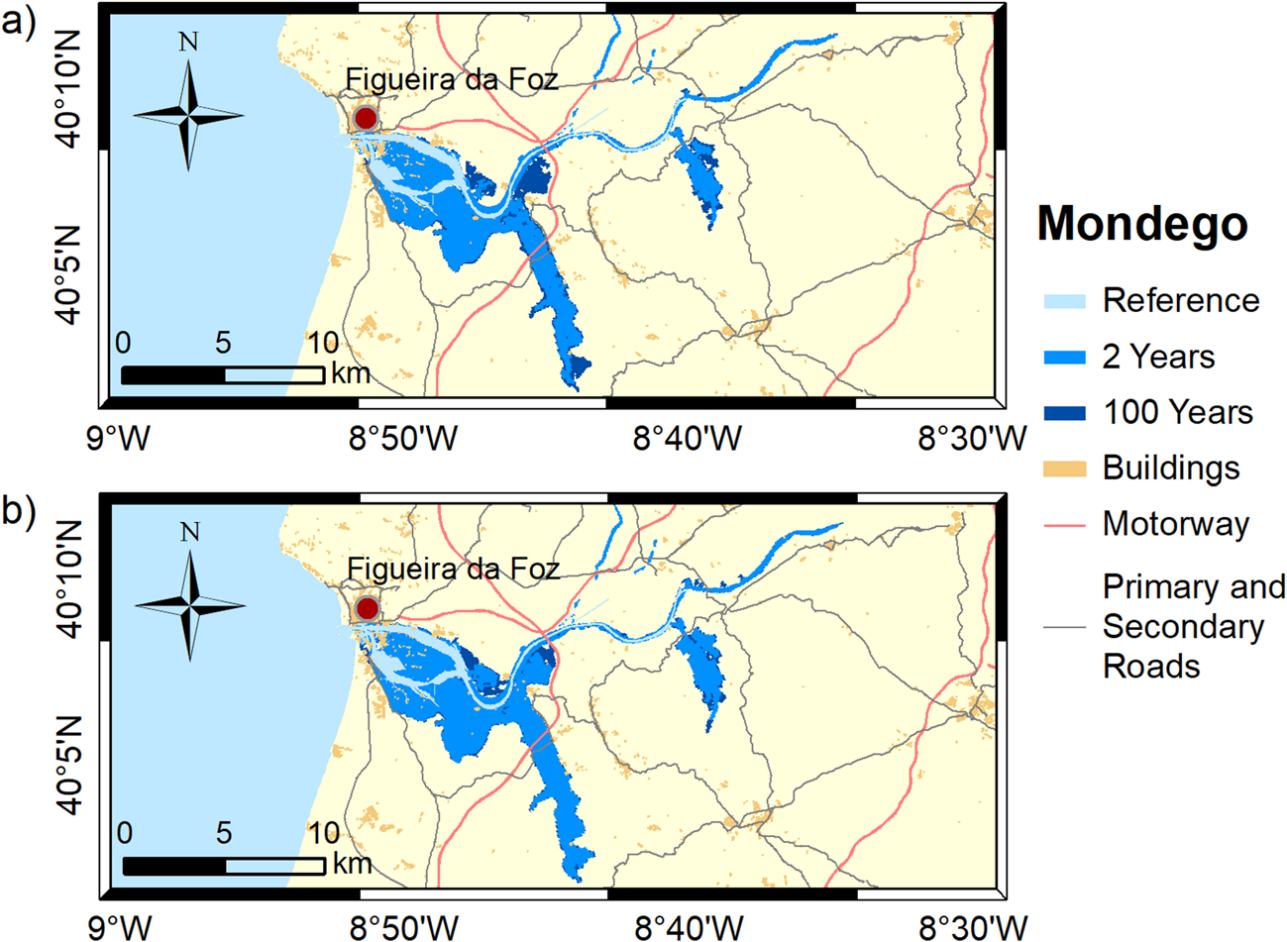
Inundation extents induced by extreme sea levels with return periods of 2 and 100 years on the Mondego estuary in: (**a**) 2046–2065; and (**b**) 2081–2100. These maps were created with Esri ArcGIS 10.8 software (https://www.esri.com/en-us/home).

**Figure 5:**
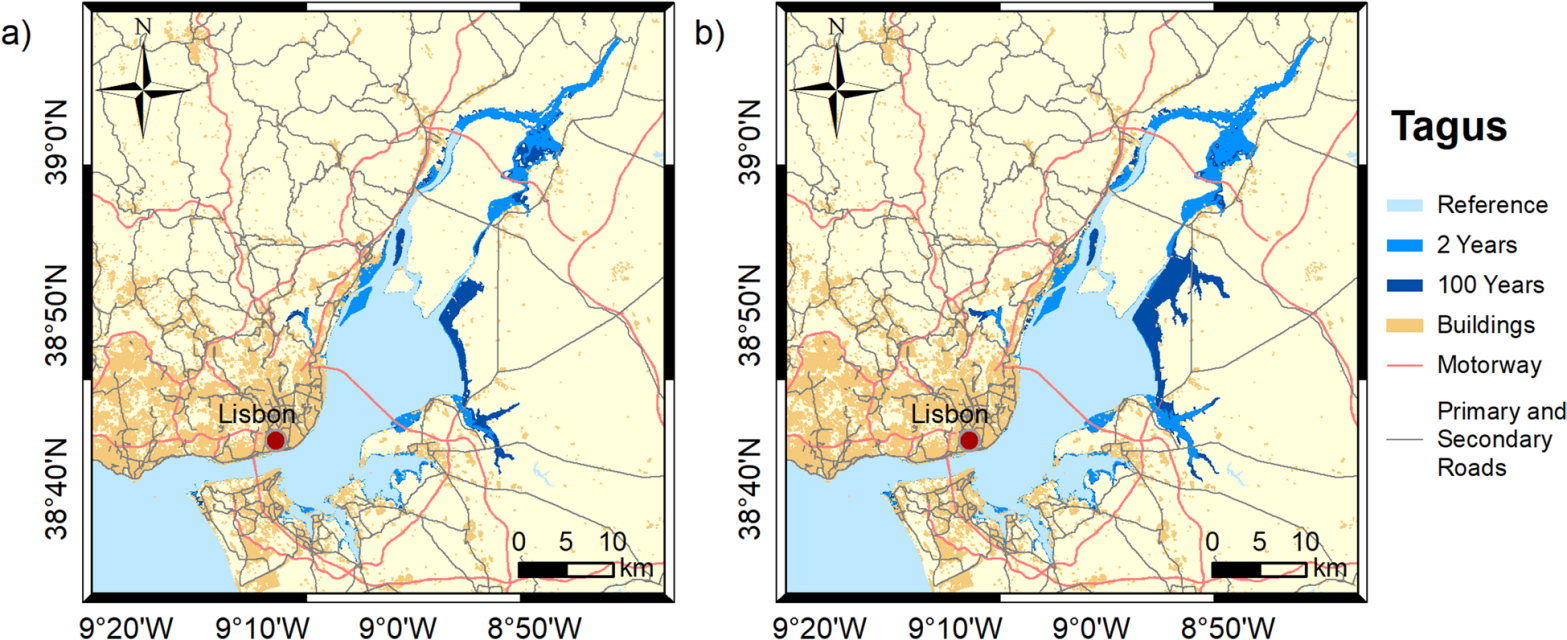
Inundation extents induced by extreme sea levels with return periods of 2 and 100 years on the Tagus estuary in: (**a**) 2046–2065; and (**b**) 2081–2100. These maps were created with Esri ArcGIS 10.8 software (https://www.esri.com/en-us/home).

**Figure 6:**
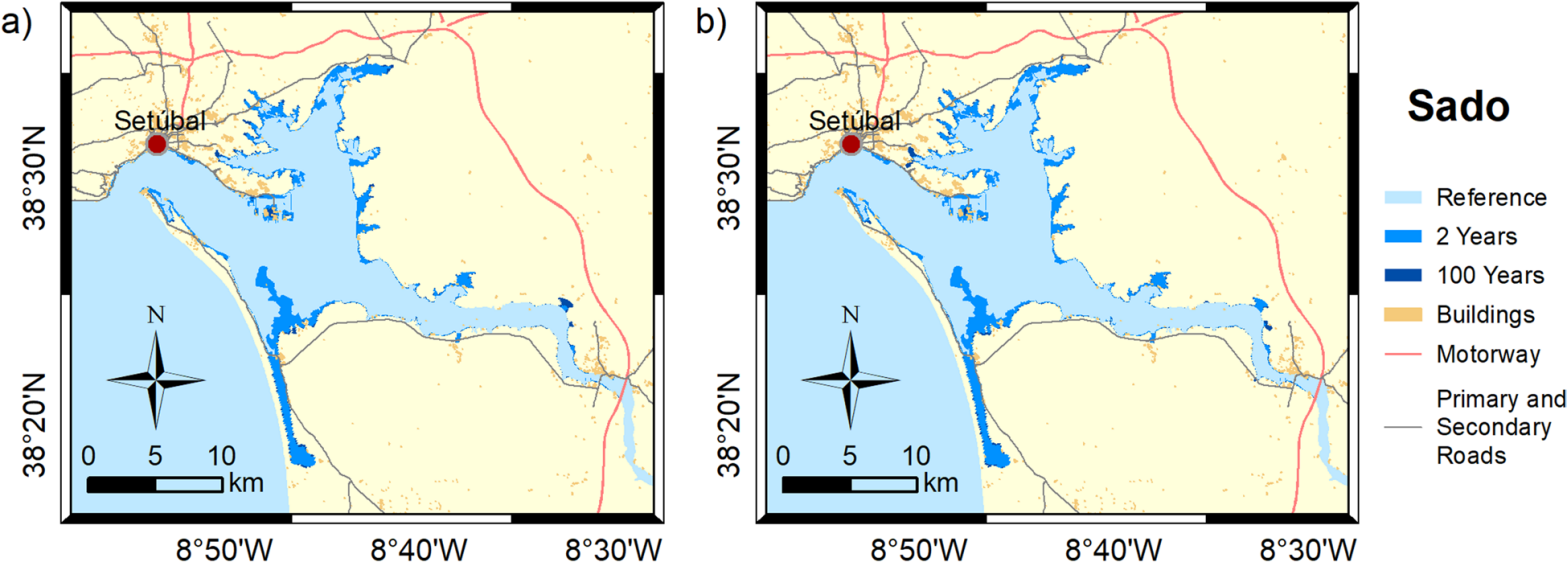
Inundation extents induced by extreme sea levels with return periods of 2 and 100 years on the Sado estuary in: (**a**) 2046–2065; and (**b**) 2081–2100. These maps were created with Esri ArcGIS 10.8 software (https://www.esri.com/en-us/home).

**Figure 7:**
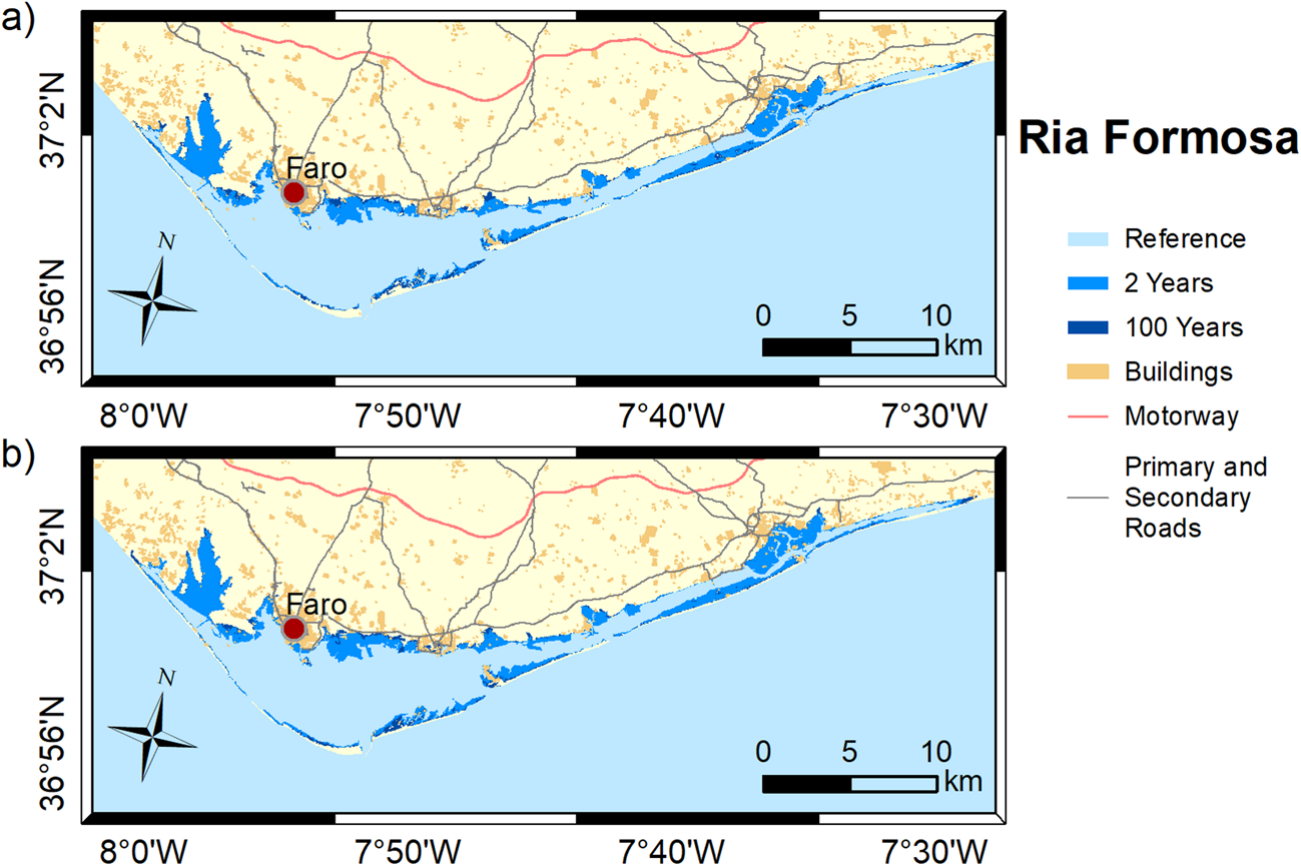
Inundation extents induced by extreme sea levels with return periods of 2 and 100 years on the Ria Formosa in: (**a**) 2046–2065; and (**b**) 2081–2100. These maps were created with Esri ArcGIS 10.8 software (https://www.esri.com/en-us/home).

Figures 3, 4, 5, 6 and 7 show the inundation extent maps, and Table 3 presents the maximum inundation area obtained for each estuarine system during the simulated extreme sea level scenarios (see “Methods” section). The Tagus is the estuary that presents the highest inundation extent (between 390 and 470 km^2^), with flooding of extensive regions to the east being observed, while Mondego is the estuary that presents the lowest inundation areas, above 60 km^2^ for all scenarios. The Tagus estuary shows the highest increases in flooding area among the scenarios under analysis, with differences of 45 and 63 km^2^ between events with a recurrence interval of 2 and 100 years, for 2046–2065 and 2081–2100, respectively. In Ria de Aveiro, these differences decrease by 21 and 19 km^2^, while in the remaining estuaries, they do not exceed 10 km^2^.

**Table 3:** Flooded area (km^2^) predicted for each scenario for each estuarine system.

	2046–2065		2081–2100	
	2 years	100 years	2 years	100 years
Ria de Aveiro	179	200	190	209
Mondego	46	55	53	59
Tagus	390	435	410	473
Sado	232	240	237	244
Ria Formosa	116	124	121	128

It is noteworthy that the extension of the flood with a recurrence period of 100 years for the medium-term horizon (2046–2065) is close to that predicted for events with a recurrence period of 2 years in the long-term horizon (2081–2100). This means that coastal floods expected every 100 years in 2046–2065 could occur every 2 years near the end of the century (2081–2100).

**Figure 8:**
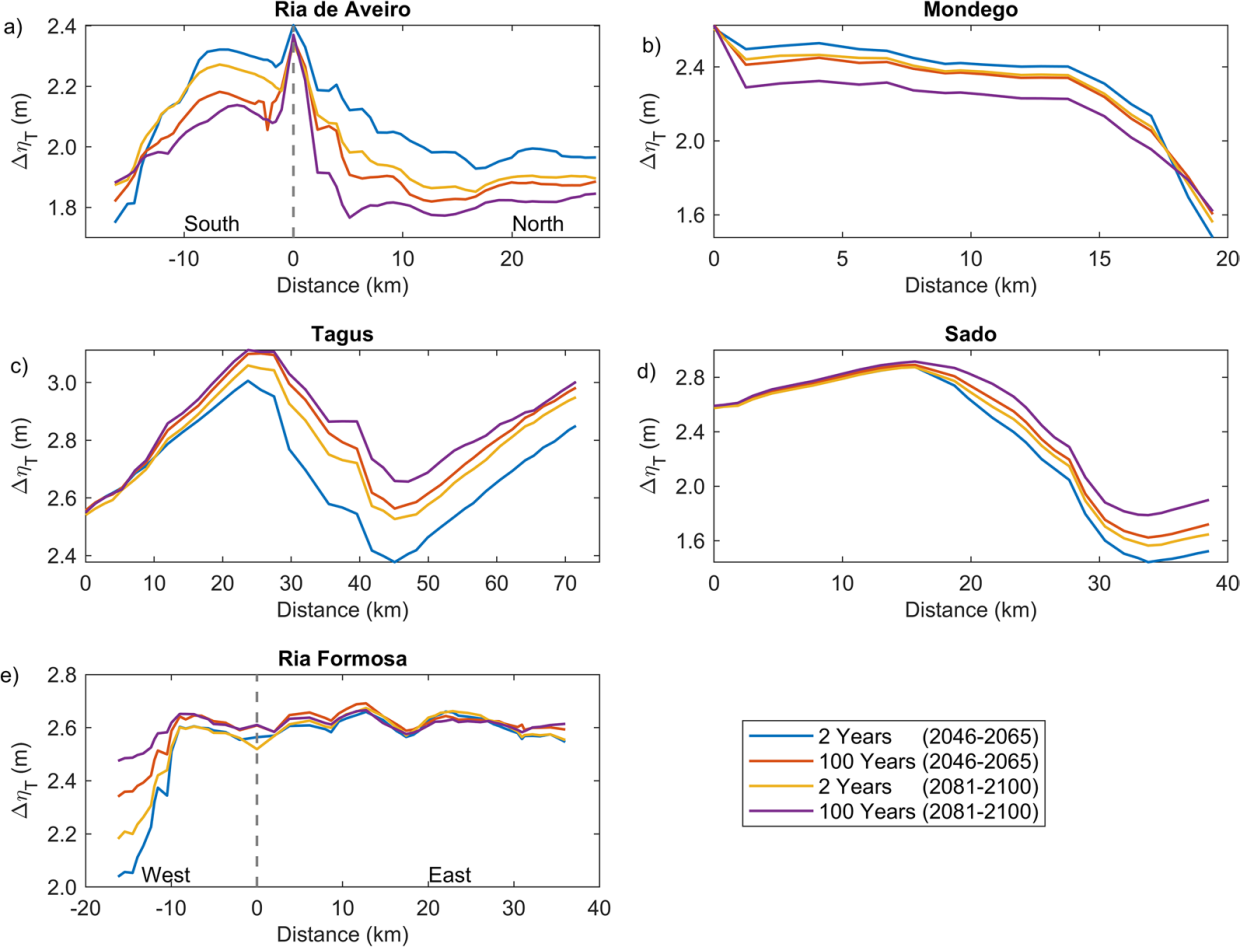
Variation of the water level range *(*$\Delta \eta_{T}$*)* along a transect starting at the estuary or lagoon mouth: (**a**) Ria de Aveiro, (**b**) Mondego, (**c**) Tagus, (**d**) Sado and (**e**) Ria Formosa. Each transect is represented by pink dots in supplementary Figs. S1, S2, S3, S4 and S5.

Figure 8 represents the evolution of the water level range (combining tides and storm surges) along transects defined in each estuary (see Supplementary Figs. S1–S5) for each extreme sea level scenario. The water level range ($\Delta \eta_{T}$), defined as the difference between the maximum water level and the next low water level (see “Methods” section), provides information about the amplification or attenuation of the long-wave as it propagates within each estuarine system. In Ria de Aveiro (Fig. 8a), the $\Delta \eta_{T}$ is largest (2.4 m for all scenarios) at the mouth and suddenly decreases in the inner lagoon (first 2 km). After that point, and moving northward (S. Jacinto channel), the $\Delta \eta_{T}$ reduces more slowly until the 9–10 km, and then stabilizes until the end of the transect. The pattern of $\Delta \eta_{T}$ reduction is also evident when moving southward (Mira channel) and is more pronounced after the 7–8 km. The results also show that the reduction in $\Delta \eta_{T}$ increases as the inundation area becomes larger. In the Mondego estuary (Fig. 8b), $\Delta \eta_{T}$ is also reduced as the flood propagates upstream, and this reduction is more pronounced after the 14 km. It is also verified that the reduction in $\Delta \eta_{T}$ is more pronounced when extreme sea levels are high. In the Tagus estuary (Fig. 8c), $\Delta \eta_{T}$ evolves differently along the transect: it amplifies from the inlet up to 25 km and then attenuates until the 45 km, amplifying again from that point to the end of the channel. The wave amplification/attenuation identified in the lower/upper estuary tends to increase/decrease as sea level rises. In Sado estuary (Fig. 8d), the wave slightly amplifies (0.20 m) from the entrance until the 15 km and after that point is found a damping that can exceed 1 m, depending on the extreme sea level scenario. The amplification in the lower estuary is similar for all scenarios, while the damping in the upper estuary is less pronounced when the extreme sea levels are high. In Ria Formosa (Fig. 8e), the values of $\Delta \eta_{T}$ are similar in most of the domain, independently of the extreme sea level scenario considered. The exception is a region in the western part that experiences a reduction in $\Delta \eta_{T}$, which is more pronounced under low inundation scenarios.

**Figure 9:**
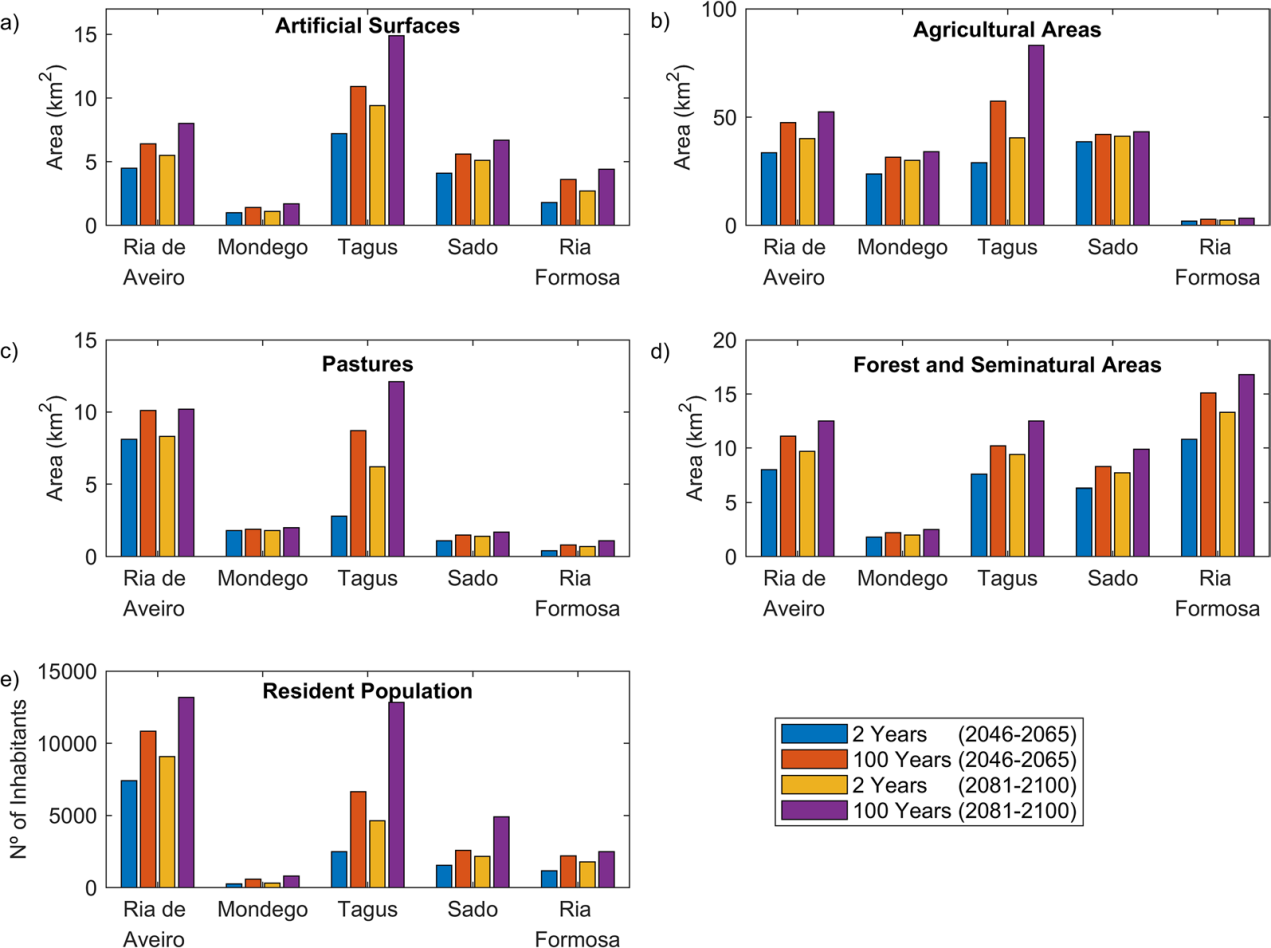
Inundation areas of different land uses: (**a**) Artificial surfaces; (**b**) Agricultural areas; (**c**) Pastures; (**d**) Forest and Seminatural areas. (**e**) Number of inhabitants directly affected by each inundation event.

Figure 9 shows the areas exposed to flooding for different land use categories and the number of residents directly affected by each flood event. In the Ria de Aveiro, Tagus, Sado and Mondego estuarine systems, floods mostly affect agricultural areas (63%, 65%, 73% and 85% of the total area, respectively), while in Ria Formosa, the category *Forest and Seminatural Areas* is the most affected (68%). It is noteworthy that the Tagus and the Ria de Aveiro will be the regions with the largest extents of agricultural areas affected by floods in the future. It is estimated that the agricultural area directly affected by events with a return period of 100 years would increase from 57.4 to 83.2 km^2^ and from 47.5 to 52.4 km^2^, respectively, between 2046–2065 and 2081–2100. The Tagus, Ria de Aveiro and Sado are the regions that present the highest areas of *Artificial Surfaces* inundated (14.9 km^2^, 8.0 km^2^ and 6.7 km^2^, respectively, for the worst scenario), while Ria Formosa and Mondego are the least affected regions (4.4 km^2^ and 1.7 km^2^, respectively, for the worst scenario). Floods with a 100 years return period could directly impact almost 23,000 and 35,000 residents by the medium and long-term horizon, respectively. Of these, 77% reside in Ria de Aveiro and Tagus adjacent regions (51% and 26%, respectively), while the remaining 23% inhabit the margins of Sado, Ria Formosa and Mondego (with 12%, 9% and 2%, respectively).

## Discussion

The accurate evaluation of extreme sea levels is a key factor when assessing flooding in coastal environments since inundation extent is generally very sensitive to local sea levels. The extreme sea levels obtained in this study evidence an increase from south to north along the Portuguese Coast, which agrees with a previous study performed by Fortunato et al.^31^. Moreover, the estimates obtained for Lagos and Cascais are slightly lower (lower by 0.08 m) than those obtained for the same locations by Antunes et al.^26^. The methodology followed by Antunes et al.^26^ is also based on the statistical analysis of tide gauge data, however, when joining the astronomical and meteorological components, it assumes the maximum tidal level instead of the level of MHWS.

The scientific community is unanimous about the continued rise in mean sea level on the Portuguese Coast until 2100, however there are a wide variety of projections. By extrapolating the mean sea level rise rate derived for the Cascais tidal gauge, Antunes and Taborda^32^ estimated a mean rise of 0.47 m by 2100, compared to 1990. In turn, it was projected by Lopes et al.^33^ a mean rise of 0.42 m for 2100 by analyzing the GISS-ER model outputs for the Portuguese Coast under the Special Report on Emissions Scenarios (SRES) A2 defined in the fourth IPCC report. These estimates are considerably lower than the 1.14 m, with a likely range between 0.39 and 1.89 m, obtained for 2100 by Antunes^34^ for Cascais considering the mean sea level rise acceleration during the twenty-first century, which was described through 2nd order polynomial functions. This study reports that the mean sea level could increase 0.68 m by 2081–2100 compared to 1986–2005, which corresponds to the median of the likely range. This value exceeds in 0.21 and 0.26 m the projections derived by Antunes and Taborda^32^ and Lopes et al.^33^, respectively. The highest projections relative to the studies of Antunes and Taborda^32^ and Lopes et al.^33^ were expected since the first study does not consider any acceleration in mean sea level rise, and the estimate derived in the second study is sustained on SRESA2, that presents a lower radiative forcing than RCP 8.5^35^. The projection presented in this study is within the range obtained by Antunes^34^ but exceeds the median value by 0.49 m, in part because the projection of this author considers the value for 2100 instead of the mean value for the interval 2081–2100 (which would decrease by 0.15 m). Although the consistency of the projection obtained here with previous studies, it is important to note that considering the decadal average instead of the 20-year period and the 5th and 95th percentile instead of the median value, leads to rises of 0.50 and 1.35 m by 2100 for Cascais under SPP5-8.5 (https://sealevel.nasa.gov/ipcc-ar6-sea-level-projection-tool).

The results obtained in this study demonstrate that flooding in coastal environments is highly dependent on the magnitude of extreme sea levels and on the geomorphology of estuarine environments, which control the flow propagation and determine the amplification or damping of the propagating long-wave. In Ria de Aveiro the inundation area varies significantly with extreme sea level changes since the low elevation of floodplains. The inlet geometry and the inundation of extensive low-lying areas revealed the main factors affecting the long-wave propagation. The long-wave is strongly damped at the lagoon entrance due to inlet constraints. This finding is supported by an inlet choking number of 3.7, which was determined through the formulation of Hill^36^, and considering an average inlet depth, width and length of 16 m, 300 m and 2500 m, respectively. According to Hill^36^, when the inlet chocking number is below 5, choking becomes important, and long-wave amplitudes strongly decrease within the embayment. The long-wave continues to damp along the inner lagoon due to energy dissipation when: (1) propagating throughout shallow channels (depth lower than 1 m); and (2) flooding low-lying lands (75% de lagoon area is tidal flats^37^). The Mondego is the estuary that presents the smallest variation in the inundated area between the extreme sea level scenarios evidencing also that the flooded barriers are very efficient in preventing the flooding of the low-lying areas adjacent to the upper estuary. In this case, the inlet chocking number is 9.1 (6 m of average depth, 180 m wide and 1500 m long) and therefore, the long-wave is not as attenuated across the inlet as in the Ria de Aveiro. The effect of energy dissipation is also evident in the inner estuary associated with the flooding of low-lying areas (tidal flats and adjacent low-lying lands), which represent around 50% (approximately 28 km^2^) of the total inundated area. The inundation extent of the Tagus estuary varies considerably between extreme sea levels, showing that the flood barriers installed in the margins of the upper estuary are ineffective in preventing the inundation of adjacent low-lying regions under the most severe extreme sea level scenarios. Contrary to Ria de Aveiro and Mondego estuary, the long-wave amplifies at the inlet and lower estuary due to the resonance effect that amplifies the semidiurnal constituents^38,39^ and then dampens when propagating throughout the tidal flats (40% of the estuary's total area). The results further suggest that the increase in the inundation extent could amplify the resonance in the lower estuary, which agrees with Fortunato et al.^38^, which revealed that the tidal flats play a major role in establishing the resonance mode. In the Sado estuary, the long-wave amplifies in the lower estuary, but contrary to the Tagus estuary, the amplification does not vary with extreme sea level scenarios, as the corresponding changes in the inundation area are small. In the Ria Formosa, the inundation extent is very similar for all the extreme sea level scenarios due to the relief of the adjacent regions that prevents flooding. Moreover, and in contrast to all other estuaries under analysis, the water level range hardly varies as it propagates along the inner lagoon, except for the western part where is found a wave attenuation due to the flooding of low-lying regions. As the changes in the inundation area are very small between scenarios, the dissipation of energy by flooding the low-lying regions is very similar, however, as the water depth increases the energy dissipated by bottom friction decreases, explaining the decrease in wave height as sea level rises.

The flood patterns obtained in this study for Ria de Aveiro and Tagus are close to the ones obtained by Fortunato et al.^24^, Lopes and Dias^40^ and Fortunato et al.^23^ through hydrodynamic modeling, despite some differences in extreme sea levels scenarios, and are very different from those obtained by Antunes et al.^26^ and Kulp and Strauss^27^ using the bathtub approach (accessible through http://www.snmportugal.pt/, and https://coastal.climatecentral.org/map/, respectively). The inundation extent obtained through the bathtub approach is considerably higher than that obtained in this study for all estuaries, this increase being particularly evident in Ria de Aveiro, Mondego and Tagus estuaries, which have extensive floodplains. This fact agrees with Hoitink and Jay^41^ and Talke and Jay^42^, evidencing the major role of low-lying areas in dissipating the long-wave energy and in controlling the maximum inundation extent. The differences between bathtub approach and numerical modeling tend to be attenuated in estuarine systems with margins with significant relief, such as Sado and Ria Formosa, as the energy dissipation caused by the inundation of floodplains is reduced. The socio-economic impacts estimated in this study are considerably lower than that estimated by Antunes et al.^26^, which is in line with the differences obtained for the inundation extent. Indeed, Antunes et al.^26^ predicted that approximately 43,000 people should be affected by coastal floods in Ria de Aveiro by 2050, which is more than three times the value projected in this study.

The results obtained in this study agree with the findings of Passeri et al.^12^ and Khojasteh et al.^13^ and reinforce the importance of using numerical models when assessing estuarine inundations. Nevertheless, it is important to emphasize the limitations of this study, which may influence the results obtained for future inundation extent and respective socio-economic impacts. The extreme sea level scenarios do not account for the uncertainty in future mean sea levels and further assume that tidal and storm surge levels will remain unchanged in the future, discarding climate-induced changes in storm surges and also the effect of mean sea level rise on tidal and storm surge propagation offshore the Portuguese Coast. The later assumptions are supported by the findings of Vousdoukas et al.^43^ and Pickering et al.^44^ and may have little or no influence on the predictions obtained for the inundation extent. Vousdoukas et al.^43^ used a numerical model to simulate storm surges along Europe's coastline for 2100 under the RCP4.5 and RCP8.5 scenarios and found that the height of storm surges could decrease by 10% on the Portuguese Coast. Additionally, Pickering et al.^44^ verified through numerical modeling that the mean sea level rise would have a minor influence on the tidal propagation offshore the Portuguese Coast, estimating a decrease of 0.02 m in the mean high water level when considering a rise in mean sea level of 2 m. Otherwise, the inundation extent and the socio-economic impacts would differ if the uncertainty in future mean sea levels is considered. Indeed, using the numerical implementations developed in this study and considering a mean sea level rise of 1.35 m (95th percentile of SSP5-8.5) superimposed on a storm surge with a return period of 100 years, we obtained inundation areas of 236 km^2^, 65 km^2^, 609 km^2^, 248 km^2^ and 132 km^2^ for Ria de Aveiro, Mondego, Tagus, Sado and Ria Formosa, respectively. These areas represent an increase of 13%, 10%, 29%, 1.6% and 3% compared to the situation under analysis corresponding to the mean sea level rise of 0.68 m. Despite the moderate and small increments in the inundation extent, the number of inhabitants directly affected would increase 56% 76%, 155%, 88% and 49%, for Ria de Aveiro, Mondego, Tagus, Sado and Ria Formosa, highlighting that small increases in the inundation extent could have a huge socio-economic impact.

The numerical simulations performed in this study disregard the influence of short-waves and river discharges on estuarine hydrodynamics following the findings of previous studies, which found the minor importance of these forcing compared with tides. Vaz et al.^45^ found for the Ria de Aveiro that the influence of short-waves is restricted to the inlet area and can raise the sea levels by 0.15 m at high tide. In the Tagus estuary, the short-waves are dissipated at the entrance, and the locally-generated waves may increase sea levels up to 0.05 m in most of the estuary, except for the deeper channels where significant wave heights can reach 0.8 m^46^. Attending all of this, the short-waves will have a minor impact on the inundation patterns determined in this study. Regarding rivers inflow, previous studies concluded that freshwater discharges affect the propagation of the tide throughout the estuaries, increasing the distortion and reducing the amplitude and speed, which results in a raising of the mean water levels and flood hazard^47,48^. Therefore, the inundation extent obtained here could be higher if river flows were considered, particularly in Ria de Aveiro, Mondego and Tagus estuaries given the floodplains adjacent to the river's channels. Lopes et al.^49^ and Fortunato et al.^23^ evaluated the combined effect of coastal and fluvial floods in Ria de Aveiro and Tagus, respectively, through numerical modeling and concluded that the inundation extent increases close to the mouth of the rivers due to the convergence of salt and freshwater during the tidal flooding. In Mondego, the high river inflows might cause ruptures in the dikes, as in December 2019 (https://emergency.copernicus.eu/mapping/list-of-components/EMSR417), and inundate extensive unprotected low-lying areas. It is noteworthy that the influence of river inflows may increase in the future climate during winter, given the intensification of extreme precipitation events projected for Portugal^50–52^.

This study also considers that the geomorphology of the estuaries will remain unchanged in the future, discarding the implementation of new flood protection structures and changes in the bathymetry. This is an important limitation with a considerable impact on the inundation extent reported here, however it is unfeasible to predict the medium and long-term geomorphologic changes given the uncertainty and unpredictability of future anthropogenic activities to be performed in these estuarine systems. However, given the importance of geomorphologic changes in establishing inundation patterns, future works should assess their influence on these patterns, especially in Ria de Aveiro and Tagus Estuary, which are the systems most susceptible to coastal flooding. Previous studies conducted in Ria de Aveiro reinforced the major role of the geomorphology on the tidal dynamics and inundation extent. Lopes et al.^53^ found that the inundation extent increased after the deepening of the lagoon channels (due to dredging activities) and Lopes and Dias^40^ concluded that the impact of mean sea level rise on tidal dynamics would depend on the future geomorphology.

Despite the aforementioned limitations, this study provides a comprehensive understanding of the physical mechanisms that determine the inundation extent in estuaries with distinct geomorphological features and advises the application of numerical models instead of bathtub approach, particularly when evaluating the inundation extent in estuaries that have: (1) adjacent low-lying areas; (2) choked inlets; (3) extensive tidal flats; (4) resonance conditions. In the Ebro Delta and Guadalquivir estuaries in Spain and Po Delta in Italy, the bathtub approach may overestimate the inundation extent because it does not consider the attenuation of long-wave along the estuary, nor the energy dissipation resulting from flooding the low-lying areas adjacent to the estuaries. The inundation extent in Lagos (Nigeria) and Ebrié (Ivory Coast) lagoons, and Richmond, Clarence and Macleay estuaries (Australia) may also be overestimated by the application of the bathtub approach as it disregards the attenuation of the long-wave both when crossing the narrow and shallow inlets and when propagating throughout the shallow channels and low-lying areas. In estuaries with a length close to 1/4 of the wavelength of the incident wave, resonance occurs, which translates into an amplification of the propagating long-wave^13,42^. Rising sea levels and flood protection structures may change the geometry and depth of estuaries affecting the resonance and consequently the inundation extent^13,42^. Contrary to bathtub approach, previous numerical modeling applications revealed efficient in simulating resonance in estuaries and, therefore should be used hereafter when assessing flood hazard in resonant or near resonant estuaries, such as Ems estuary (Germany and Netherlands), Elbe estuary (Germany) or Chesapeake Bay (United States of America)^54,55^.

Precise predictions of future inundation extent and the comprehensive understanding of the associated physical mechanisms within estuaries are urgent to provide stakeholders engagement and guidance on policy development. However, the increasing uncertainty on future socio-economic developments (that affect mean sea level) and anthropogenic activities within estuaries (that affect estuarine geomorphology) makes more complex and uncertain to accurately predict the future inundation extent, posing major challenges to the efficient and sustainable management of estuaries. Moreover, as flood mapping directly influences flood risk perception and public awareness, engagement and preparedness^28,30^, care must be taken when communicating flood projections and underlaying uncertainties since this influences trust in science and message acceptance^56,57^. In detail, the previous studies concluded that including uncertainty information in climate change promotes public trust in climate science, since a range of possible futures may be seen as more credible than a single, worst-case scenario.

## Methods

### Study area

The coastline of Portugal (Fig. 1) extends over approximately 1200 km and is characterized by sandy, rocky and cliffed coasts and wetlands (estuaries and lagoons). This study will analyze the Sado, Tagus and Mondego estuaries and Ria de Aveiro and Ria Formosa coastal lagoons, identified as the Portuguese coastal wetlands most threatened by climate induced mean sea level rise^26,27,58^.

Starting north on the west coast, Ria de Aveiro is a shallow lagoon with an artificial tidal inlet and four main channels: Mira, S. Jacinto, Ílhavo, and Espinheiro. Ria de Aveiro hydrodynamics is mainly regulated by the tide (oceanic forcing), and by the complex geomorphology of the lagoon^59^. At neap tide, the inundation area is 64.9 km^2^ which increases to 89.2 km^2^ at spring tide^60^. Southward, with an area of 8.6 km^2^^61^ it is located the Mondego estuary. This estuary is characterized by two water arms (north and south arm) separated by Murraceira island. These two arms have different hydrologic characteristics and a constantly changing geomorphology, due to the tidal action and the seasonal high discharges of the Mondego river^62^. The Tagus estuary has an area of 320 km^2^^63^ and is considered the most important aquatic transition system in the country, due to the high population density verified mainly close to the inlet marginal regions. The center part of the estuary is 25 km long and 15 km wide and has a complex bottom topography with tidal flat areas and small islands on the inner upper part^64^. The Sado estuary has an area of 100 km^2^^65^, and presents extensive tidal flats (one-third of its total area) characterized by mudflats and salt marshes^66^. With a domain of about 84 km^2^^67^, Ria Formosa is located on the south coast of Portugal. This lagoon is protected by a multi-inlet barrier system comprising five islands, two peninsulas, and six tidal inlets (two fixed and four natural)^68^. Like Ria de Aveiro, this shallow system also exhibits a complex network of tidal channels, and extensive tidal flats composed by salt marshes, mudflats and sand flats^69^.

The dominant mechanisms responsible for the flooding in Portuguese estuaries and lagoons are tides, storm surges and mean sea level rise^23–25,31^. Tides are predominantly semidiurnal, however the propagation of the tidal wave within each coastal system has different features. In Ria de Aveiro, Mondego and Ria Formosa, the tidal amplitude is dampened upstream and its phase increases due to the shallow depths and narrower channels they present^59,70,71^. Tagus and Sado estuaries show an opposite behavior with a slight amplification of the tidal amplitude in the inner regions of the estuaries^64,66^.

Regarding storm surges, these events occur when low pressure systems across the Atlantic nearshore Portugal. The average storm surge height is 0.45 m, however the strongest events can exceed 1 m height^72,73^.

Concerning mean sea level, the analysis of tidal gauge records in Cascais (West Coast) and Lagos (South Coast), revealed rising trends of 1.3 ± 0.1 and 1.5 ± 0.2 mm/y, respectively^74^. Recent investigations highlight that the rate of mean sea level rise accelerated in Cascais between 1996–2016 and 3.1 mm/y^34^.

A common point among these five estuarine systems that should be highlighted for their importance is that they all provide a variety of resources, benefits and services of great ecological and economic value. All of them host harbors sheltered in their inland waters that are vital for navigation and transport, playing a fundamental role in the Portuguese economy. Furthermore, some of the most densely populated Portuguese cities are located along the margins of these estuaries: Lisbon, Setúbal, Aveiro, Faro and Figueira da Foz are located in the margins of Tagus, Sado, Ria de Aveiro, Ria Formosa and Mondego, respectively, and present population densities per square kilometer of 5093.1, 498.0, 401.5, 301.3 and 155.2, respectively (https://www.pordata.pt/en/Municipalities/Population+density-452). It is noteworthy that the margins of Tagus, Ria de Aveiro and Mondego show extensive agricultural fields of vital importance to local populations and the national economy.

### Extreme sea levels

Extreme sea levels on the Portuguese Coast were assessed by analyzing hourly sea levels recorded, between 1976–2019, 1960–2019 and 1986–2019 in Aveiro, Cascais and Lagos, respectively (see location in Fig. 1). The tide gauge records of Aveiro were made available by the Authority of the Aveiro Harbour (APA) and those of Cascais and Lagos by the University of Hawaii Sea Level Center (UHSLC) through https://uhslc.soest.hawaii.edu/^75^and the Directorate-General for the Territory (DGT), respectively.

For each site, the sea level annual records were decomposed into astronomic and residual levels by performing tidal harmonics using t_tide package^76^. The astronomic levels were then used to determine the level of MHWS over 18.6 years, while storm surge events were detected and characterized by processing the residual series with the algorithm developed by Pinheiro et al.^73^. It is noteworthy that extreme sea levels were determined when tidal levels are higher than MHWS, as recommended by Pugh (2004). A joint probability approach was applied to tide and storm surge data for determining extreme sea levels for return periods of 2, 10, 25, 50 and 100 years.

The climate induced mean sea level changes were evaluated by analyzing the sea-level projection data from the Intergovernmental Panel on Climate Change (IPCC) 5th and 6th Assessment Report (AR5 and AR6) for the Portuguese Coast. Data from AR5 was provided by the Integrated Climate Data Center (ICDC) of the University of Hamburg through the LAS (Live Access Server) interface (https://icdc.cen.uni-hamburg.de/1/daten/ocean/ar5-slr.html). This data has 1° of spatial resolution and corresponds to the ensemble mean from a set of 21 Atmosphere–Ocean General Circulation Models (AOGCM). The sea level change spatial variability was investigated on the Portuguese Coast considering the Representative Concentration Pathway (RCP) 4.5 and 8.5, which are representative of moderate and pessimistic stabilizations of radiative forcing, respectively. Data from AR6 was obtained through the NASA Sea Level Projection Tool (https://sealevel.nasa.gov/ipcc-ar6-sea-level-projection-tool), which provides local projections for the five Shared Socioeconomic Pathway (SSP) scenarios defined in the AR6. In detail, projections of mean sea level change were extracted and analyzed for each decade covering the period 2020–2100 for Cascais station under scenarios SSP2-4.5 and SSP5-8.5. SSP2-4.5, commonly termed *“Middle of the road”,* assumes a balance between mitigation and adaptation, while SSP5-8.5 is characterized by low mitigation and high adaptation^77^. It is important to highlight that scenarios RCP4.5 and RCP8.5 slightly differ from SSP2-4.5 and SSP5-8.5, respectively, regarding radiative forcing^77^. Sea level time series projections were compared with mean sea levels derived from tide gauges observations in order to verify the consistency between observations and projections. Finally, mean sea level rise projections by 2046–2065 and 2081–2100 were determined for scenarios RCP4.5, RCP8.5, SSP2-4.5 and SSP5-8.5.

### Models implementation and validation

The numerical model used to characterize the inundation extent for Ria de Aveiro, Mondego, Tagus, Sado and Ria Formosa estuarine systems is the Delft3D-Flow^78^. This is an open-source three-dimensional finite-difference hydrodynamic code developed by WL|Delft Hydraulic in cooperation with the Delft University of Technology. The model solves the 3D baroclinic Navier–Stokes and transport equations under the Boussinesq assumption.

Numerical domains were defined through curvilinear meshes generated for the five estuarine systems under study (Table 4). These grids adopt spherical coordinates and include the estuarine channels, intertidal areas, adjacent floodplains, and margins with elevations lower than 8 m. The depth interpolation for each grid element considered the aggregation of bathymetric and topographic data taken from several databases: Ria de Aveiro was obtained from Polis Litoral Ria de Aveiro, S.A and Aveiro Port Administration; Mondego and Ria Formosa were provided by Hydrographic Institute of the Portuguese Navy; and Tagus and Sado were obtained from compilation of data surveys collected between 1964 and 2009. The values corresponding to the intertidal regions, floodplains and margins were obtained using metadata with a resolution of 1 m resulting from the application of LIDAR technology in 2011, provided by the General Direction of the Territory.

**Table 4:** Main parameters of the Delft3D numerical simulations.

Estuarine system	Grid domain		Bottom roughness
	Dimensions (MxN)	Average resolution (m)	
Ria de Aveiro	1042 × 397	50	Lopes et al.^53^
Mondego	569 × 729	50	Lopes et al.^53^
Tagus	724 × 1744	50	Ribeiro et al.^65^
Sado	622 × 568	100	Ribeiro et al.^65^
Ria Formosa	1624 × 432	40	Dias et al.^70^

Thirteen main tidal harmonic constants (M_2_, S_2_, N_2_, K_2_, K_1_, O_1_, P_1,_ Q_1_, MF, MM, M_4_, MS_4_, MN_4_) obtained from the model TPXO 7.2 TOPEX/Poseidon Altimetry (http://volkov.oce.orst.edu/tides/global.html) were used as the astronomical forcing at the oceanic open boundary of the model.

A spatial variable friction coefficient was assumed for bottom roughness for the five estuarine systems. The values were chosen considering the studies performed in previous works (Table 4). At the margins and floodplains, the Manning coefficients are dependent on the Corine Land Cover data set of 2006^79^ and were attributed according to Lopes et al.^60^.

The model time step is 15 s for the Ria de Aveiro and 30 s for the remaining estuarine systems. These time steps were chosen after sensitivity tests to determine the largest time step for which the model still gives accurate results. The numerical implementations adopt an Alternating Direction Implicit (ADI) time integration method that guarantees accurate results for Courant numbers lower than $4\sqrt 2$^78^. The maximum Courant number achieved during numerical simulations was 3.4, which guarantees the stability and accuracy of numerical results. For all estuarine systems, the horizontal eddy viscosity was set to 1 m^2^ s^−1^. All simulations performed considered a space varying Coriolis force calculated from the latitude coordinates in the grid files.

The numerical implementations adopt a wetting and drying algorithm to accurately represent the covering and uncovering of the tidal flats and the flooding of marginal regions. In detail, the algorithm removes the grid cells that become dry during the ebb and adds grid cells that become wet during the flood. The method to decide if a grid cell is wet or dry is based on a user defined threshold depth. A cell is assigned wet when the water depth exceeds the threshold and dry when the water depth is less than half of the threshold. The five numerical implementations considered a threshold depth of 0.01 m, estimated from a thumb rule that considers the tidal amplitude and the total number of time steps during a tidal cycle^78^.

The effect of flood barriers on the flooding was represented through the sub-grid process weir 2D^78^. Weirs are fixed structures with a user defined crest height considered in the wetting and drying algorithm to determine if a velocity point is wet or dry. The numerical implementations of Ria de Aveiro, Mondego and Tagus adopt weir 2D to represent existent roads and dikes, which crest height was attributed according to topographic data available (supplementary Figs. S1, S2 and S3). This process was not considered in Sado and Ria Formosa due to the lack of flood protection structures in these coastal systems.

The model validation was evaluated through a qualitative and quantitative comparison of the temporal evolution of model results of sea surface elevation, and concurrent in-situ data sampled during 30 days for the Ria de Aveiro, Tagus, Sado and Ria Formosa and a tidal cycle for the Mondego estuary (Fig. S1, supplementary material). The validation was performed by adjusting the bottom friction coefficient and viscosity for all estuarine systems. The root mean square error (*RMSE*) and predictive skill was computed for the stations shown in Figs. S1, S2, S3, S4 and S5 (supplementary material) to quantify the model accuracy in reproducing in-situ data.

Table 5 summarizes the model accuracy in reproducing observed sea surface elevation. In general, it was found an adequate fit between model results and observations, revealing the ability of the models to accurately reproduce *in-*situ data. The average *RMSE* value is 0.13 m for Ria de Aveiro, 0.14 m for Mondego, 0.10 m for Tagus and 0.07 m for Sado and Ria Formosa. The most accurate model results were obtained for the stations near the estuarine system mouth, indicated by the lower *RMSE* values, and the highest disagreements were found for the innermost parts. Predictive skills are between 0.973 and 0.999 for all domains (not shown in Table 5). The *RMSE* and predictive skill values obtained are very similar to those obtained in previous numerical modeling works^65,71,73,80^ showing an excellent accuracy of the hydrodynamic models developed in reproducing the long-wave propagation.

**Table 5:** RMSE (m) for the estuarine systems under study.

Station	Ria de Aveiro	Mondego	Tagus	Sado	Ria Formosa
1	0.05	0.10	0.05	0.08	0.07
2	0.07	0.09	0.07	0.05	0.08
3	0.18	0.23	0.06	0.07	0.07
4	0.13	0.12	0.08	0.07	0.12
5	0.14	–	0.09	0.07	0.06
6	0.07	–	0.14	0.07	0.07
7	0.19	–	0.15	–	0.05
8	0.16	–	0.11	–	0.05
9	0.07	–	0.17	–	–
10	–	–	0.10	–	–
11	–	–	0.09	–	–
12	–	–	0.07	–	–
13	–	–	0.07	–	–

The numbers represent the stations shown in Fig. S1 of the supplementary material.

### Inundation mapping and population and asset exposure

Once validated, the numerical models were used to predict the maximum inundation extent for the four scenarios. For each location, sea levels time series were generated to impose as ocean boundary condition in the model. These time series result from the superposition of the astronomical tide, storm surge and mean sea levels (supplementary Fig. S6). The astronomical tide was generated through harmonic synthesis considering the same tidal constants considered in validation simulations (TPXO 7.2 TOPEX/Poseidon Altimetry). The storm surge levels were generated artificially using a sine function and considering that the storm surge persists for three days. When adding the time series, the storm surge peak and the level of MHWS were made coincident. Attending the location of tidal gauges and estuarine systems, the time series generated for Aveiro were used to force the implementations of Ria de Aveiro and Mondego estuary, those obtained for Cascais and Lagos were used to force the implementations of Tagus and Sado Estuaries and Ria Formosa. Noteworthy is the rigor followed with this methodology that considers the best local forcing conditions, aiming to obtain results with the maximum possible accuracy.

For all scenarios, the model is run for ten days, followed by the determination of the maximum inundation extent from the analysis of the sea surface elevation and the maximum inundation area that was determined by adding the area of the grid cells that are flooded at least once during the simulation. The sea surface levels ($\eta \left( x \right)$) were further used to assess the water level range ($\Delta \eta_{T}$) during the storm surge event, according to:

$$\Delta \eta_{T} = \mathop {\max }\limits_{t} \left( {\eta \left( x \right)} \right) - \mathop {\min }\limits_{t} \left( {\eta \left( x \right)} \right)$$

The evolution of $\Delta \eta_{T}$ throughout each study area was analyzed for all flooding scenarios.

Inundation extent was transposed into a Geographic Information System (GIS) framework and intersected with georeferenced socio-economic data of the study region. Socio-economic data include the Census of 2011 Cartographic Base (BGRI 2011) provided by Statistics Portugal (INE) and the Land Use Chart of 2018 produced and delivered by the DGT. It is noteworthy that the BGRI 2011 census data was disaggregated into a local scale. The number of inhabitants directly affected by floods and the areas exposed to flooding for different land use types were determined and analyzed for each study site and each scenario.

## Supplementary Information

Supplementary Information.

## Data Availability

The data that support the findings of this study are available from the corresponding author upon reasonable request.

## References

[1] Clark KE, Niles LJ, Burger J (1993). Abundance and distribution of migrant shorebirds in Delaware Bay. Condor.

[2] Roman CT, Jaworski N, Short FT, Findlay S, Warren RS (2000). Estuaries of the northeastern United States: Habitat and land use signatures. Estuaries.

[3] Bergstrom JC, Dorfman JH, Loomis JB (2004). Estuary management and recreational fishing benefits. Coast. Manag..

[4] Vousdoukas, M. I. *et al. *Adapting to Rising Coastal Flood Risk in the EU Under Climate Change. https://ec.europa.eu/jrc (2020) 10.2760/456870.

[5] Weisse R (2014). Changing extreme sea levels along European coasts. Coast. Eng..

[6] Teng J (2017). Flood inundation modelling: A review of methods, recent advances and uncertainty analysis. Environ. Model. Softw..

[7] Nkwunonwo UC, Whitworth M, Baily B (2020). A review of the current status of flood modelling for urban flood risk management in the developing countries. Sci. Afr..

[8] Apel H, Aronica GT, Kreibich H, Thieken AH (2009). Flood risk analyses: How detailed do we need to be?. Nat. Hazards.

[9] Kumbier K, Carvalho RC, Vafeidis AT, Woodroffe CD (2018). Investigating compound flooding in an estuary using hydrodynamic modelling: A case study from the Shoalhaven River Australia. Hazards Earth Syst. Sci.

[10] Didier D (2019). Multihazard simulation for coastal flood mapping: Bathtub versus numerical modelling in an open estuary Eastern Canada. J. Flood Risk Manag..

[11] Ramirez JA, Lichter M, Coulthard TJ, Skinner C (2016). Hyper-resolution mapping of regional storm surge and tide flooding: Comparison of static and dynamic models. Nat. Hazards.

[12] Passeri DL (2015). The dynamic effects of sea level rise on low-gradient coastal landscapes: A review. Earth’s Futur..

[13] Khojasteh D, Glamore W, Heimhuber V, Felder S (2021). Sea level rise impacts on estuarine dynamics: A review. Sci. Total Environ..

[14] Didier D (2015). Coastal flood assessment based on field debris measurements and wave runup empirical model. J. Mar. Sci. Eng..

[15] Gallien TW, Sanders BF, Flick RE (2014). Urban coastal flood prediction: Integrating wave overtopping, flood defenses and drainage. Coast. Eng..

[16] Vousdoukas MI (2016). Developments in large-scale coastal flood hazard mapping. Nat. Hazards Earth Syst. Sci..

[17] Gallien TW (2016). Validated coastal flood modeling at Imperial Beach, California: Comparing total water level, empirical and numerical overtopping methodologies. Coast. Eng..

[18] Seenath A, Wilson M, Miller K (2016). Hydrodynamic versus GIS modelling for coastal flood vulnerability assessment: Which is better for guiding coastal management?. Ocean Coast. Manag..

[19] Kirezci E (2020). Projections of global-scale extreme sea levels and resulting episodic coastal flooding over the 21st Century. Sci. Rep..

[20] Hanslow DJ, Morris BD, Foulsham E, Kinsela MA (2018). A Regional scale approach to assessing current and potential future exposure to tidal inundation in different types of estuaries. Sci. Rep..

[21] Neumann, T. & Ahrendt, K. *Comparing the ‘Bathtub Method’ with MIKE 21 HD Flow Model for Modelling Storm Surge Inundation: Case Study Fiel Fjord*. (2013).

[22] Kumbier K, Carvalho RC, Vafeidis AT, Woodroffe CD (2019). Comparing static and dynamic flood models in estuarine environments: A case study from south-east Australia. Mar. Freshw. Res..

[23] Fortunato AB (2017). A numerical study of the February 15, 1941 storm in the Tagus estuary. Cont. Shelf Res..

[24] Fortunato AB, Rodrigues M, Dias JM, Lopes C, Oliveira A (2013). Generating inundation maps for a coastal lagoon: A case study in the Ria de Aveiro (Portugal). Ocean Eng..

[25] Lopes CL, Dias JM (2015). Assessment of flood hazard during extreme sea levels in a tidally dominated lagoon. Nat. Hazards.

[26] Antunes C, Rocha C, Catita C (2019). Coastal flood assessment due to sea level rise and extreme storm events: A case study of the Atlantic coast of Portugal’s mainland. Geosciences.

[27] Kulp SA, Strauss BH (2019). New elevation data triple estimates of global vulnerability to sea-level rise and coastal flooding. Nat. Commun..

[28] Zabini F, Grasso V, Crisci A, Gozzini B (2021). How do people perceive flood risk? Findings from a public survey in Tuscany Italy. J. Flood Risk Manag..

[29] Houston D (2017). The influence of hazard maps and trust of flood controls on coastal flood spatial awareness and risk. Perception.

[30] Moon T (2020). Ending a sea of confusion: Insights and opportunities in sea-level change communication. Environ.: Sci. Policy Sustain. Develop..

[31] Fortunato AB, Li K, Bertin X, Rodrigues M, Miguez BM (2016). Determination of extreme sea levels along the Iberian Atlantic coast. Ocean Eng..

[32] Antunes, C. & Taborda, R. Sea level at cascais tide gauge: Data, analysis and results. *J. Coast. Res.* 218–222 (2009).

[33] Lopes CL (2011). Local sea level change scenarios for the end of the 21st century and potential physical impacts in the lower ria de aveiro (Portugal). Cont. Shelf Res..

[34] Antunes C (2019). Assessment of sea level rise at west coast of Portugal mainland and its projection for the 21st century. J. Mar. Sci. Eng..

[35] Collins M, Stocker TF (2013). Long-term Climate Change: Projections, Commitments and Irreversibility. Climate Change 2013 The Physical Science Basis Contribution of Working Group I to the Fifth Assessment Report of the Intergovernmental Panel on Climate Change.

[36] Hill AE (1994). Fortnightly tides in a lagoon with variable choking. Estuar. Coast. Shelf Sci..

[37] Silveira F, Lopes CL, Pinheiro JP, Pereira H, Dias JM (2021). Coastal floods induced by mean sea level rise&mdash;ecological and socioeconomic impacts on a mesotidal lagoon. J. Mar. Sci. Eng.

[38] Fortunato AB, Oliveira A, Baptista AM (1999). On the effect of tidal flats on the hydrodynamics of the Tagus estuary. Oceanol. Acta.

[39] Dias JM, Valentim JM, Sousa MC (2013). A numerical study of local variations in tidal regime of tagus estuary Portugal. PLoS One.

[40] Lopes CL, Dias JM (2015). Tidal dynamics in a changing lagoon: Flooding or not flooding the marginal regions. Estuar. Coast. Shelf Sci..

[41] Hoitink AJF, Jay DA (2016). Tidal river dynamics: Implications for deltas. Rev. Geophys..

[42] Talke SA, Jay DA (2020). Changing tides: The role of natural and anthropogenic factors. Ann. Rev. Mar. Sci..

[43] Vousdoukas MI, Voukouvalas E, Annunziato A, Giardino A, Feyen L (2016). Projections of extreme storm surge levels along Europe. Clim. Dyn..

[44] Pickering MD (2017). The impact of future sea-level rise on the global tides. Cont. Shelf Res..

[45] Vaz L, Plecha S, Dias JM (2013). Coastal wave regime influence on Ria de Aveiro inlet dynamics. J. Coast. Res.

[46] Rusu L, Bernardino M, Guedes Soares C (2011). Modelling the influence of currents on wave propagation at the entrance of the Tagus estuary. Ocean Eng..

[47] Xiao Z (2021). Characterizing the non-linear interactions between tide, storm surge, and river flow in the Delaware Bay Estuary United States. Front. Mar. Sci..

[48] Spicer P, Huguenard K, Ross L, Rickard LN (2019). High-frequency tide-surge-river interaction in estuaries: Causes and implications for coastal flooding. J. Geophys. Res. Ocean..

[49] Lopes CL, Alves FL, Dias JM (2017). Flood risk assessment in a coastal lagoon under present and future scenarios: Ria de Aveiro case study. Nat. Hazards.

[50] Soares PMM, Cardoso RM, Ferreira JJ, Miranda PMA (2015). Climate change and the Portuguese precipitation: ENSEMBLES regional climate models results. Clim. Dyn..

[51] Cardoso Pereira S, Marta-Almeida M, Carvalho AC, Rocha A (2020). Extreme precipitation events under climate change in the Iberian Peninsula. Int. J. Climatol..

[52] Santos M, Fonseca A, Fragoso M, Santos JA (2019). Recent and future changes of precipitation extremes in mainland Portugal. Theor. Appl. Climatol..

[53] Lopes CL, Plecha S, Silva PA, Dias JM (2013). Influence of morphological changes in a lagoon flooding extension: Case study of Ria de Aveiro (Portugal). J. Coast. Res..

[54] Lee SB, Li M, Zhang F (2017). Impact of sea level rise on tidal range in Chesapeake and Delaware Bays. J. Geophys. Res. Ocean..

[55] Hein SSV, Sohrt V, Nehlsen E, Strotmann T, Fröhle P (2021). Tidal oscillation and resonance in semi-closed estuaries: Empirical analyses from the elbe estuary North Sea. Water.

[56] Retchless DP (2014). Sea level rise maps: How individual differences complicate the cartographic communication of an uncertain climate change hazard. Cartogr. Perspect..

[57] Howe LC, MacInnis B, Krosnick JA, Markowitz EM, Socolow R (2019). Acknowledging uncertainty impacts public acceptance of climate scientists’ predictions. Nat. Clim. Chang..

[58] Ferreira Ó, Dias JA, Taborda R (2008). Implications of sea-level rise for continental Portugal. J. Coast. Res..

[59] Dias JM, Lopes JF, Dekeyser I (2000). Tidal propagation in Ria de Aveiro lagoon, Portugal. Phys. Chem. Earth, Part B Hydrol. Ocean. Atmos..

[60] Lopes CL, Azevedo A, Dias JM (2013). Flooding assessment under sea level rise scenarios: Ria de Aveiro case study. J. Coast. Res..

[61] Teixeira H, Salas F, Borja Á, Neto JM, Marques JC (2008). A benthic perspective in assessing the ecological status of estuaries: The case of the Mondego estuary (Portugal). Ecol. Indic..

[62] Flindt MR (1997). Description of the three shallow estuaries: Mondego River (Portugal), Roskilde Fjord (Denmark) and the Lagoon of Venice (Italy). Ecol. Modell..

[63] Fortunato AB, Oliveira A, Baptista AM (1999). On the effect of tidal flats on the hydrodynamics of the Tagus estuary. Oceanol. Acta.

[64] Fortunato A, Baptista AM, Luettich RA (1997). A three-dimensional model of tidal currents in the mouth of the Tagus estuary. Cont. Shelf Res..

[65] Ribeiro AS, Sousa MC, Lencarte Silva JD, Dias JM (2016). David and Goliath revisited: Joint Modelling of the tagus and sado estuaries. J. Coast. Res..

[66] Martins F, Leitão P, Silva A, Neves R (2001). 3D modeling in the Sado estuary using a new generic vertical discretization approach. Oceanol. Acta.

[67] de Andrade, C. A. C. F. O ambiente de barreira da Ria Formosa: Algarve-Portugal. (Universidade de Lisboa, 1990).

[68] Salles P (2001). Hydrodynamic Controls on Multiple Tidal Inlet Persistence.

[69] Pacheco A, Ferreira Ó, Carballo R, Iglesias G (2014). Evaluation of the production of tidal stream energy in an inlet channel by coupling field data and numerical modelling. Energy.

[70] Dias, J. M. & Sousa, M. C. Numerical modeling of Ria Formosa tidal dynamics. in *Journal of Coastal Research (Proceedings of the 10th International Coastal Symposium)* 1345–1349 (2009).

[71] Mendes J (2021). Modeling dynamic processes of mondego estuary and óbidos lagoon using delft3D. J. Mar. Sci. Eng..

[72] Picado A, Lopes CL, Mendes R, Vaz N, Dias JM (2013). Storm surge impact in the hydrodynamics of a tidal lagoon: the case of Ria de Aveiro. J. Coast. Res..

[73] Pinheiro JP, Lopes CL, Ribeiro AS, Sousa MC, Dias JM (2020). Tide-surge interaction in Ria de Aveiro lagoon and its influence in local inundation patterns. Cont. Shelf Res..

[74] Dias, A. & Taborda, R. Tidal gauge data in deducing secular trends of relative sea level and crustal movements in Portugal. *J. Coast. Res.* vol. 8 https://journals.flvc.org/jcr/article/view/78897 (1992).

[75] Caldwell, P. C., Merrifield, M. A. & Thompson, P. R. Sea level measured by tide gauges from global oceans: The Joint Archive for Sea Level holdings (NCEI Accession 0019568). *NOAA Natl. Centers Environ. Inf.* **version 5.**, Dataset (2015).

[76] Pawlowicz R, Beardsley B, Lentz S (2002). Classical tidal harmonic analysis including error estimates in MATLAB using TDE. Comput. Geosci..

[77] Chen D., Masson-Delmotte V (2021). Framing, Context, and Methods. in Climate Change 2021: The Physical Science Basis. Contribution of Working Group I to the Sixth Assessment Report of the Intergovernmental Panel on Climate Change.

[78] Deltares. *Delft3D-FLOW User Manual*. (2020).

[79] EEA. *CLC2006 technical guidelines*. (2007).

[80] Dias JM, Sousa MC, Bertin X, Fortunato AB, Oliveira A (2009). Numerical modeling of the impact of the Ancao Inlet relocation (Ria Formosa, Portugal). Environ. Model. Softw..

