# Supplementary material for: Evaluation of future estuarine floods in a sea level rise context
Source: Sci Rep. 2022 May 16;12:8083. doi: 10.1038/s41598-022-12122-7 (PMC9110397; doi:10.1038/s41598-022-12122-7)
Supplement: Supplementary file 1 — Supplementary Information. [file 41598_2022_12122_MOESM1_ESM.pdf]

A Reliable Evaluation of Future Estuarine Floods in a Sea Level Rise Context

Carina Lurdes Lopes[1]*, Magda Sousa[1], Américo Ribeiro[1], Humberto Pereira[1], João Pedro Pinheiro[1], Leandro Vaz[1] & João Miguel Dias[1]

[1] CESAM—Centre for Environmental and Marine Studies, Physics Department, University of Aveiro, Aveiro, Portugal. *email: carinalopes@ua.pt

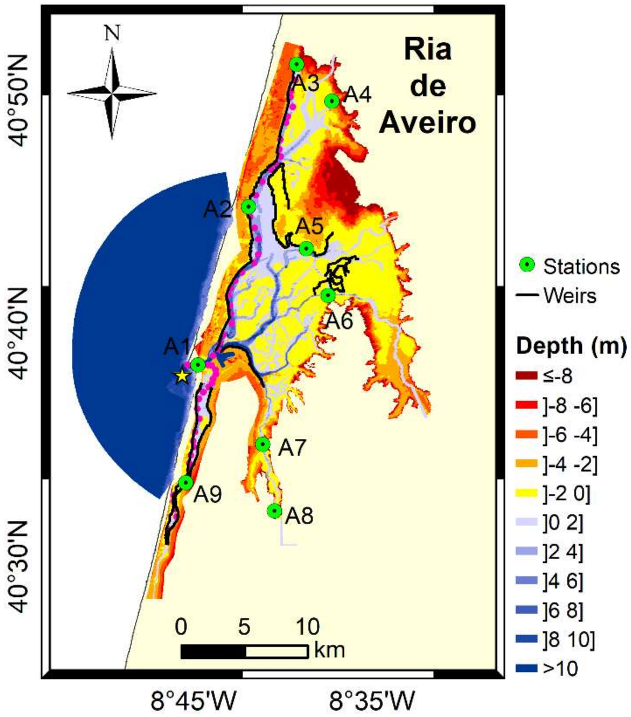

Figure S1- *Numerical bathymetry of Ria de Aveiro. The black lines represent the flood-barriers and the green dots the location of stations used to validate the hydrodynamic model. The pink dots represent the segment with the origin at the lagoon mouth (yellow star) in which maximum and minimum levels were analyzed. This map was created with Esri ArcGIS 10.8 software (https://www.esri.com/en-us/home).*

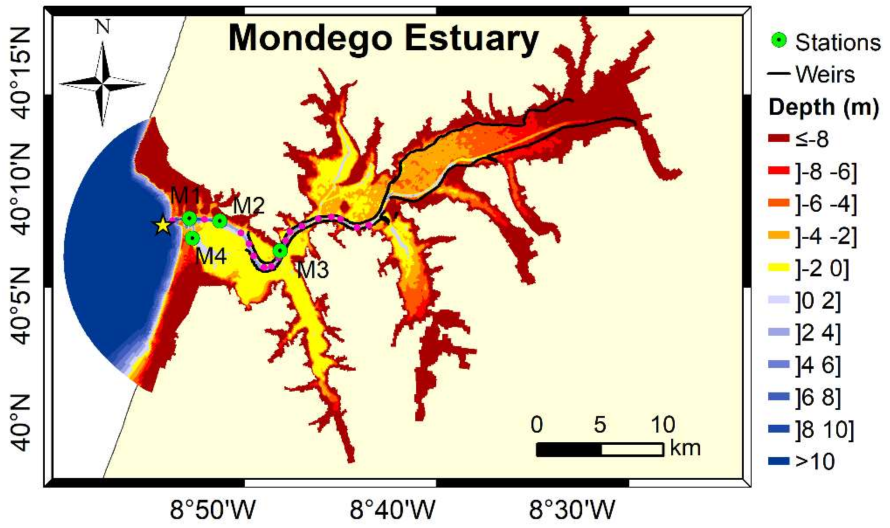

Figure S2- *Numerical bathymetry of Mondego Estuary. The black lines represent the flood-barriers and the green dots the location of stations used to validate the hydrodynamic model. The pink dots represent the segment with the origin at the estuary mouth (yellow star) in which maximum and minimum levels were analyzed. This map was created with Esri ArcGIS 10.8 software (https://www.esri.com/en-us/home).*

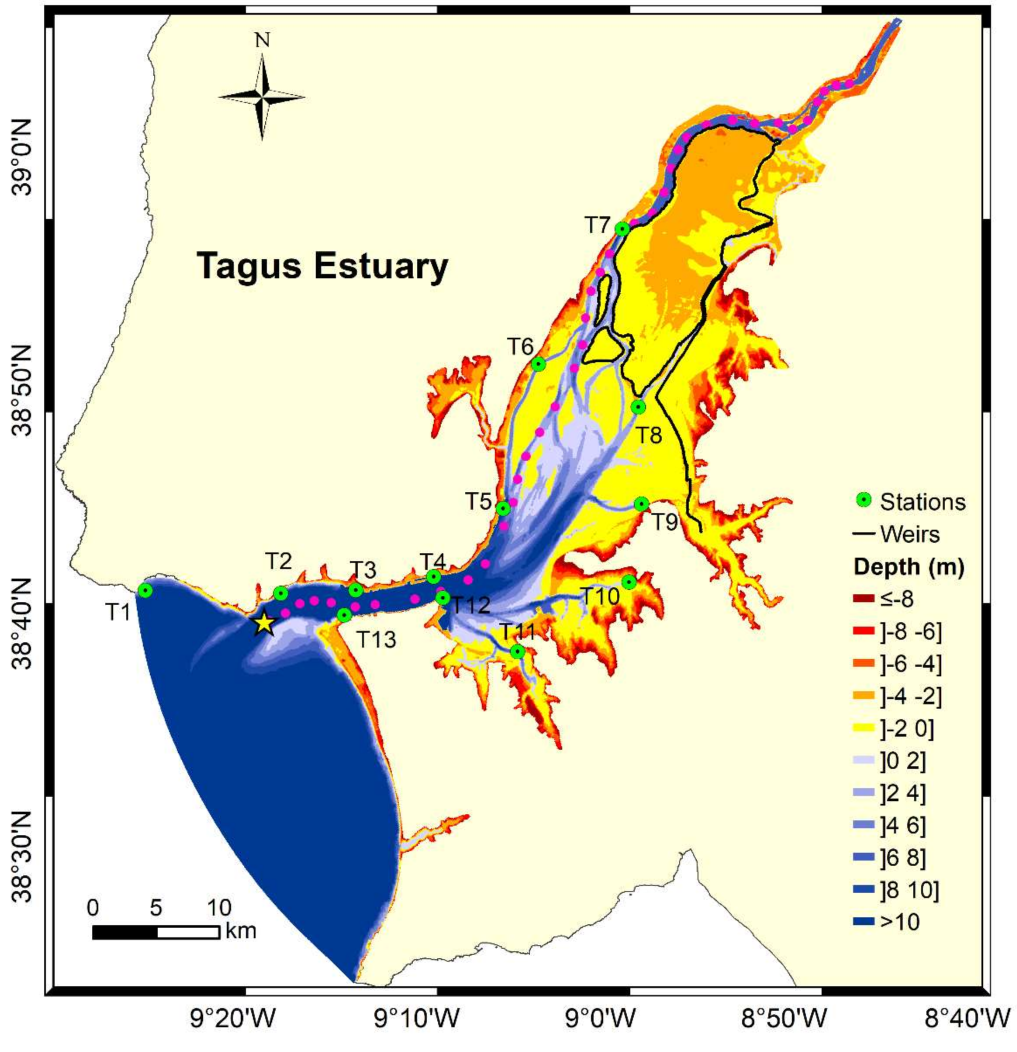

Figure S3- *Numerical bathymetry of Tagus Estuary. The black lines represent the flood-barriers and the green dots the location of stations used to validate the hydrodynamic model. The pink dots represent the segment with the*

origin at the estuary mouth (yellow star) in which maximum and minimum levels were analyzed. This map was created with Esri ArcGIS 10.8 software (https://www.esri.com/en-us/home).

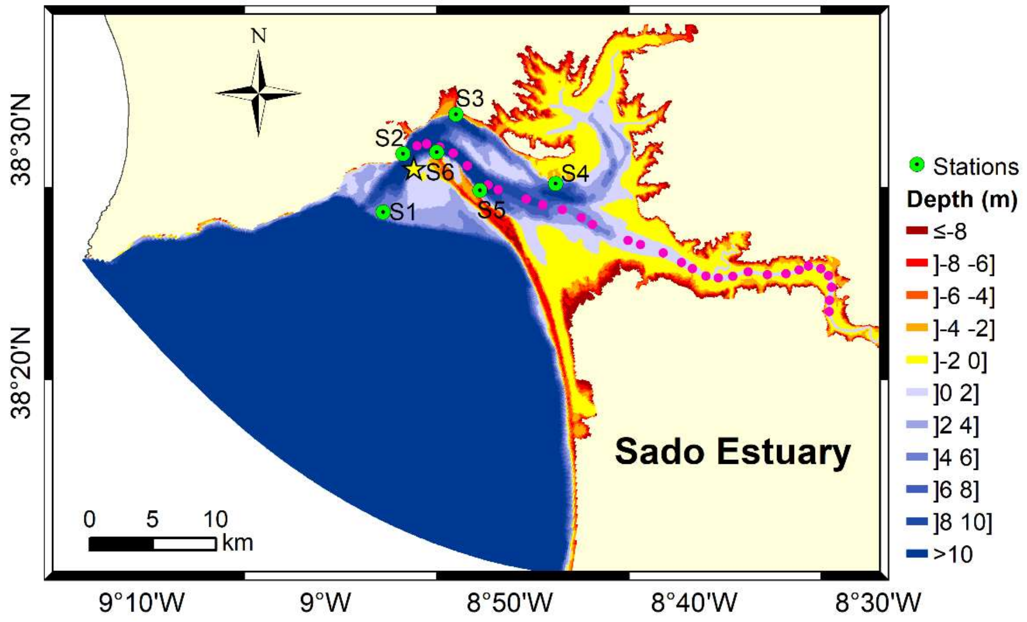

***Figure S4**- Numerical bathymetry of Sado Estuary. The green dots show the location of stations used to validate the hydrodynamic model. The pink dots represent the segment with the origin at the estuary mouth (yellow star) in which maximum and minimum levels were analyzed. This map was created with Esri ArcGIS 10.8 software (https://www.esri.com/en-us/home).*

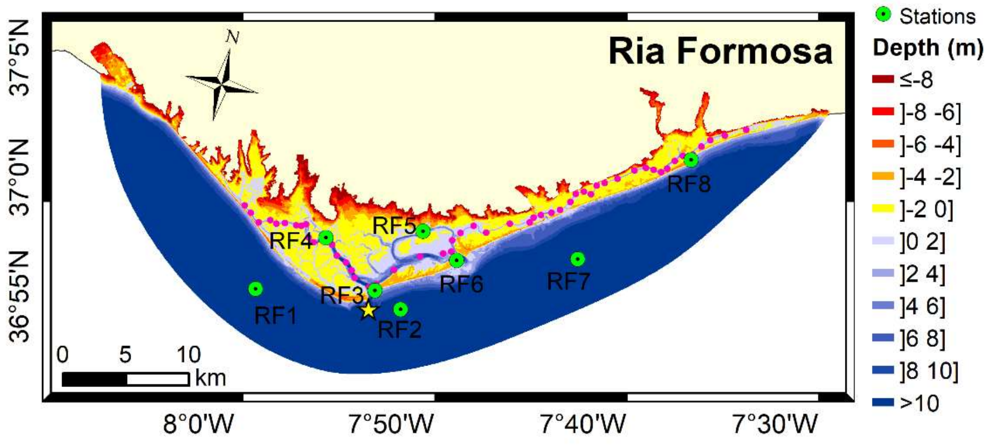

***Figure S5**- Numerical bathymetry of Ria de Aveiro. The green dots show the location of stations used to validate the hydrodynamic model. The pink dots represent the segment with the origin at one the lagoon mouths (yellow star) in which maximum and minimum levels were analyzed. This map was created with Esri ArcGIS 10.8 software (https://www.esri.com/en-us/home).*

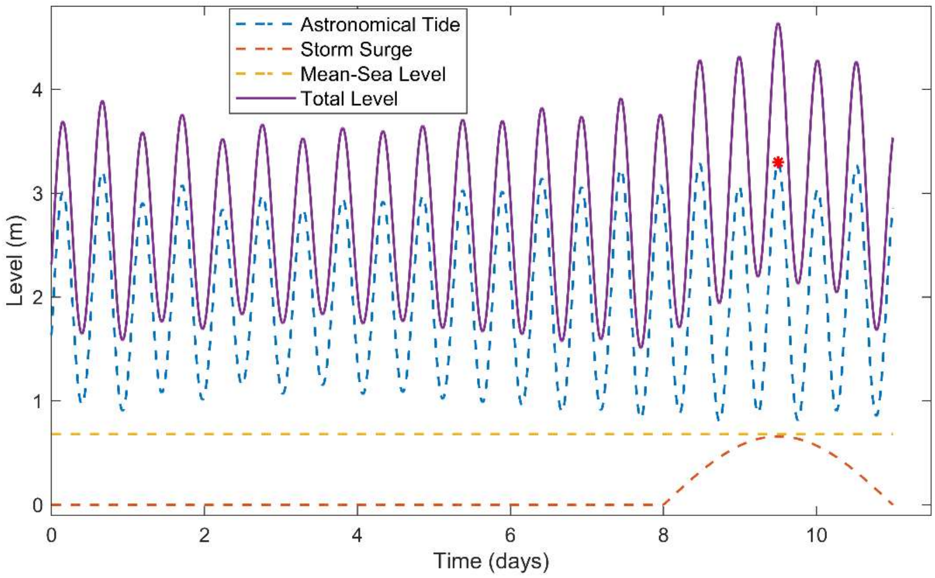

Figure S6- *Time series of sea surface levels (m) imposed as boundary condition. Dashed curves represent the level of each component. The red mark represents the level of MHWS.*
